# The Superior Adherence Phenotype of E. coli O104:H4 is Directly Mediated by the Aggregative Adherence Fimbriae Type I

**DOI:** 10.1080/21505594.2020.1868841

**Published:** 2021-01-15

**Authors:** Philipp Schiller, Michael Knödler, Petya Berger, Lilo Greune, Angelika Fruth, Alexander Mellmann, Petra Dersch, Michael Berger, Ulrich Dobrindt

**Affiliations:** a Institute of Hygiene, University of Münster, Münster, Germany; b Institute for Infectiology, University of Münster, Münster, Germany; c Division of Enteropathogenic Bacteria and Legionella, Robert Koch Institute, Wernigerode, Germany

**Keywords:** Enteroaggregative *E. coli*, enterohemorrhagic *E. coli*, AAF, adherence, biofilm, autoaggregation

## Abstract

Whereas the O104:H4 enterohemorrhagic *Escherichia coli* (EHEC) outbreak strain from 2011 expresses aggregative adherence fimbriae of subtype I (AAF/I), its close relative, the O104:H4 enteroaggregative *Escherichia coli* (EAEC) strain 55989, encodes AAF of subtype III. Tight adherence mediated by AAF/I in combination with Shiga toxin 2 production has been suggested to result in the outbreak strain’s exceptional pathogenicity. Furthermore, the O104:H4 outbreak strain adheres significantly better to cultured epithelial cells than archetypal EAEC strains expressing different AAF subtypes. To test whether AAF/I expression is associated with the different virulence phenotypes of the outbreak strain, we heterologously expressed AAF subtypes I, III, IV, and V in an AAF-negative EAEC 55989 mutant and compared AAF-mediated phenotypes, incl. autoaggregation, biofilm formation, as well as bacterial adherence to HEp-2 cells. We observed that the expression of all four AAF subtypes promoted bacterial autoaggregation, though with different kinetics. Disturbance of AAF interaction on the bacterial surface via addition of α-AAF antibodies impeded autoaggregation. Biofilm formation was enhanced upon heterologous expression of AAF variants and inversely correlated with the autoaggregation phenotype. Co-cultivation of bacteria expressing different AAF subtypes resulted in mixed bacterial aggregates. Interestingly, bacteria expressing AAF/I formed the largest bacterial clusters on HEp-2 cells, indicating a stronger host cell adherence similar to the EHEC O104:H4 outbreak strain. Our findings show that, compared to the closely related O104:H4 EAEC strain 55989, not only the acquisition of the Shiga toxin phage, but also the acquisition of the AAF/I subtype might have contributed to the increased EHEC O104:H4 pathogenicity.

## Introduction

The outbreak of enterohemorrhagic *Escherichia coli* (EHEC) O104:H4 in 2011 in Germany was one of the largest and most severe EHEC outbreaks in recent history with almost 4000 infections resulting in more than 50 fatalities [1]. Since then, research was focused on the strain that caused this unprecedented rate of infection, especially as it affected mainly healthy adults, which is not common for EHEC epidemics [2]. The severity of symptoms and the high percentage of patients developing hemolytic uremic syndrome (HUS) were attributed to the unusual combination of virulence factors of different *E. coli* pathotypes found in this strain [1,3]. Typical EHEC markers include the Shiga toxin (Stx)- and the IrgA homologue adhesin-encoding determinants *stx2* and *iha*, respectively, whereas the most prominent virulence markers of enteroaggregative *E. coli* (EAEC) include the global virulence regulator AggR-encoding and aggregative adherence fimbriae (AAF)-encoding determinants *aggR* and *aggDCBA* [4]. Whole genome analysis revealed a very close relationship of the EHEC O104:H4 outbreak strain with the prototypic EAEC isolate 55989, which was originally isolated from an HIV-positive patient in the Central African Republic with persistent diarrhea [5–8]. However, the subtype of the main virulence factor of EAEC, the AAF adhesin, was different in EHEC O104:H4 (AAF/I) when compared to EAEC strain 55989 (AAF/III) [9].

AAF were proposed to have significantly contributed to the exceptional pathogenicity of the outbreak strain, e.g. by increasing the amount of Stx2 molecules absorbed through the colon epithelial cells by the tight adherence of a high number of bacterial cells [4,10]. Up to date, five different AAF subtypes are known. In addition to the EHEC outbreak strain [4] and a recently discovered EAEC/EHEC hybrid strain [11], AAF are limited almost exclusively to EAEC. They are typically encoded on the large EAEC virulence plasmid pAA [5,12–15] and are activated by AggR [16,17], which is a central virulence regulator in EAEC [18,19]. AAF have been described as surface structures that mediate aggregative adherence to eukaryotic cells, other bacteria, and abiotic surfaces in a characteristic “stacked-brick” or “honeycomb” adherence pattern [15].

**Figure 1.**
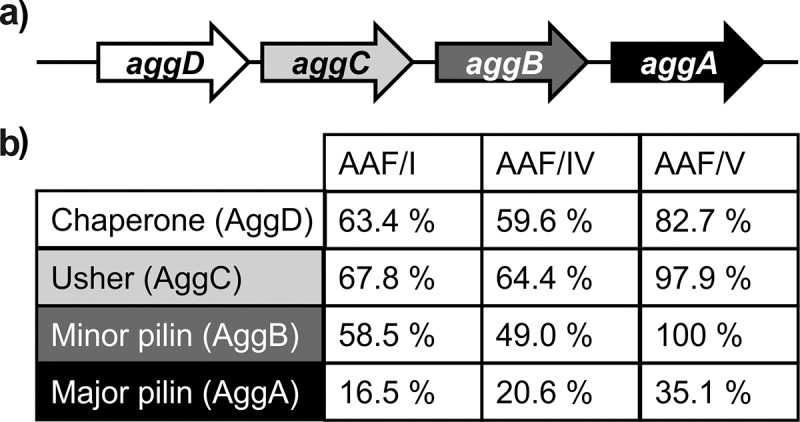
Genetic organization of the *aaf*/I, III, IV and V operons and amino acid identities of the AggDCBA subunits. (a) Schematic representation of the genetic organization of *aaf*/I, III, IV and V operons (not to scale). (b) Amino acid identities of the AggDCBA subunits of AAF/I, IV and V in respect to the subunits of AAF/III in percent. Overall, AAF/V is more closely and AAF/IV more distantly related to AAF/III than AAF/I is to AAF/III

The genes encoding AAF are usually organized in one operon that encodes four distinct proteins (schematically depicted in Figure 1(a)). The gene *aggD* encodes a chaperone protein, *aggC* encodes an usher protein, whereas the structural genes *aggA* and *aggB* encode the major and the minor fimbrial subunit, respectively [20], with AggB sitting at the tip of a polymer body formed by AggA subunits [21]. AAF are assembled using the chaperone-usher (CU) pathway, similar to various other types of fimbriae in Gram-negative bacteria [22,23]. We have recently performed a comparative characterization of virulence relevant properties of the Stx2-encoding phage-cured EHEC O104:H4 with other prototypical EAEC, including the very close relative EAEC 55989. Overall, EHEC O104:H4 displayed similar phenotypic characteristics to the other EAEC, with the notable exception that significantly more bacteria were found to be attached to cultured epithelial cells [24]. However, it was not clear, if the increased numbers of bacteria attached to cultured epithelial cells were due to other factors that are specifically encoded in EHEC O104:H4, differences in regulation and-/or expression of the AAF fimbriae, or if this phenotype could be attributed directly to different AAF/I properties. In order to phenotypically characterize AAF/III in comparison with AAF/I, we heterologously expressed AAF/I and AAF/III in AAF-negative EAEC 55989 derivative (EAEC 55989 *agg*3^−^) as well as AAF/IV and AAF/V, which are more distantly and more closely related to AAF/III than AAF/I, respectively. Expression of the AAF subtypes on the bacterial cell surface was confirmed by Western blot analysis and electron microscopy (EM). This further showed that the expression of all four AAF clusters resulted in autoaggregation and sedimentation of the aggregates in liquid culture, albeit at different kinetics. When incubated together, mixed aggregates were formed, but AAF-negative variants were not included, indicating that all tested AAF subtypes do not interact with other EAEC surface structures. Moreover, AAF-promoted biofilm formation by the different AAF subtypes was found to be temperature-dependent and correlated inversely with the observed autoaggregation phenotype. Finally, we show that AAF/I expressing EAEC strain 55989 *agg*3^−^ formed larger bacterial clusters on HEp-2 cells, similar to what we have previously shown for the outbreak strain EHEC O104:H4 [24].

## Materials and methods

### Bacterial strains and growth conditions

All bacterial strains used in this study are listed in Supplementary Table S1. All experiments were performed in lysogeny broth (LB) medium (tryptone: 10 g/L, yeast extract: 5 g/L, NaCl: 5 g/L). Where applicable, the growth medium was supplemented with ampicillin (100 µg/ml) and/or chloramphenicol (25 µg/ml).

#### *Construction of EAEC strain 55989* agg*3^−^*

In EAEC isolate 55,989 the gene cluster coding for AAF/III (*agg*3*DCBA*) was replaced by the chloramphenicol resistance cassette of pKD3 using the lambda Red recombination system [25]. The resulting strain *E. coli* 55989 *agg*3::*cat* was hereafter named EAEC 55989 *agg*3^−^.

### Plasmids

All plasmids used in this study are listed in Supplementary Table S2 and all primers used in this study are listed in Supplementary Table S3. The structural genes coding for the different AAF allelic variants were amplified from clinical isolates (see Supplementary Table S1) by PCR using the primers listed in Supplementary Table S3 and cloned into the expression vector pBAD24 [26]. The integrity of all cloned AAF encoding gene clusters was afterward confirmed by Sanger sequencing of overlapping segments.

#### Construction of pAAFI

The gene cluster coding for AAF/I (*aggDCBA*) was amplified by PCR using EHEC strain LB226692 plasmid DNA as template and primer pair agg_for/agg_rev. The PCR product was digested with *Kpn*I and cloned into the *Sma*I/*Kpn*I-digested pBAD24.

#### Construction of pAAFIII

The AAF/III determinant (*agg*3*DCBA*) was amplified by PCR using EAEC strain 55989 plasmid DNA as template and primer pair agg3_for/agg3_rev. The PCR product was digested with *Xba*I and *Hind*III and cloned into the *Hind*III/*Xba*I-digested pBAD24.

#### Construction of pAAFIV

The gene cluster coding for AAF/IV (*hdaDCBA*) was amplified by PCR using primer pair hda_for/hda_rev and plasmid DNA of EAEC isolate 13–00093 (O73:H18) as template. The PCR product was digested with *Nhe*I and *Hind*III and cloned the *Nhe*I/*Hind*III-digested pBAD24.

#### Construction of pAAFV

The AAF/V encoding genes (*agg5DCBA*) were amplified by PCR using plasmid DNA of the aUPEC isolate 147/06 (alternative designation 1352) as template and primer pair agg5_for/agg5_rev. The resulting PCR product was cut with *Xba*I and ligated into pBAD24 that had been digested with *Sma*I/*Xba*I.

#### Construction of pPS1 and pPS2

pUC*r-yfp* and pUC*r-cfp* were constructed as described previously, except that primers MBPD 82/83 were used for the amplification of the kanamycin resistance cassette from pACYC177 [18]. pUC*r-yfp* and pUC*r-cfp* were used as templates to generate linear PCR products to generate chromosomal P*dps-cfp* and P*dps-yfp* transcriptional fusions in *E. coli* K-12 strain MG1655 as described previously [25]. The P*dps-cfp* and P*dps-yfp* fusion constructs were amplified from chromosomal DNA with primers MBP 140 and MBPD 156, digested with *Kpn*I and cloned into pWKS30 [27], digested with *Fsp*I and *Kpn*I to create pPS1 (dpsP-*cfp*) and pPS2 (dpsP-*yfp*), respectively, for the differential fluorescent labeling of stationary phase *E. coli* cells.

### Western blot analysis

10 ml of induced overnight cultures (LB medium supplemented with arabinose 1% (w/v)) was harvested by centrifugation (10 min, 3000 x g, 4°C). The supernatant was discarded, and the bacterial pellets were washed by re-suspending in sterile PBS followed by centrifugation for 10 min at 3000 x g and 4°C. The washing step was repeated twice. Pellets were frozen for 30 min at −80°C and then resuspended in 1.1 ml protein resuspension buffer (1045 µl PBS, 11 µl PMSF solution (100 mM), 44 µl protein inhibitor complete (25x) (Roche, Mannheim, Germany). The samples were transferred to Lysing Matrix E tubes (MP Biomedicals, Eschwege, Germany) and processed with the Precellys24 tissue homogenizer (Bertin Technologies, Montigny-le-Bretonneux, France). The supernatants were transferred to new tubes and stored at −20°C until further use. The protein concentrations of the whole cell extracts were determined using the Protein Assay Dye Reagent Concentrate (BioRad, Munich, Germany) according to the manufacturer’s instructions in a Tecan Infinite F200 plate reader (Tecan, Crailsheim, Germany). 10 µg, 5 µg, and 2.5 µg of total protein extracts of EAEC strain 55989 *agg3^−^* harboring one of the plasmids pAAF/I, pAAF/III, pAAF/IV or pAAF/V, as well as 10 µg of EAEC strain 55989 *agg*3^−^ (pBAD24) were subjected to SDS-PAGE and Western blotting according to the standard protocols described elsewhere [28]. Polyclonal antibodies used for detection of AAF variant major subunits AggA to Agg5A expression were generated by Davids Biotechnologie GmbH (Regensburg, Germany). Primary antibodies were used in a 1:2000 dilution. For the detection of the primary antibodies an anti-rabbit antibody coupled with horseradish peroxidase (HRP) (Dianova, Hamburg, Germany) (1:15,000) and the Clarity ECL solution (BioRad, Munich, Germany) was used. The signals were recorded using the ChemiDoc MP System (BioRad, Munich, Germany). For details about antibodies see Supplementary Table S4.

### Autoaggregation assay

EAEC 55989 *agg*3^−^ cells containing either pBAD24 or the plasmids encoding the different AAF subtypes were plated on LB agar containing the suitable antibiotics and incubated overnight at 37°C. Afterward, a single colony was inoculated in 2 ml LB medium containing the suitable antibiotics and grown overnight at 37°C and 180 rpm. The next day, 100 µl of these pre-cultures were used to inoculate 20 ml fresh LB medium and the cultures were grown at 37°C and 180 rpm. The OD_600_ was determined using the Ultraspec 2100pro spectrophotometer (Amersham Biosciences, Freiburg, Germany) in regular intervals until an OD_600_ of 0.4–0.6 had been reached. 600 µl of a 10% arabinose solution were added and incubation continued for another 60 min. Sterile glass tubes were filled with 5 ml bacterial culture and kept static at room temperature. For the measurements, 100 µl samples were taken approximately 1 cm below the liquid surface in duplicates and the OD_600_ was determined. Samples were taken every 10 min in the first 60 min after the beginning of the experiment and afterward every 60 min for a total assay time of 3 h.

To test if the addition of purified specific AAF antibodies to the bacterial suspension disturbs AAF interaction and thus bacterial autoaggregation, we prepared the autoaggregation assays with EAEC strain 55989 *agg*3^−^ expressing the different AAF subtypes as described above. We then added 25 µl of purified antibodies raised against the individual AAF subtype per ml of bacterial suspension and measured the optical density of the bacterial suspension in the absence and presence of the antibodies at time point 0 min and 60 min, respectively.

### Mixed Aggregation Assay

EAEC 55989 *agg*3^−^ cells containing either pBAD24 or the plasmids encoding the different AAF gene clusters, as well as pPS1 or pPS2 (encoding *cfp* and *yfp*, respectively) were inoculated in 2 ml LB medium with glucose (0.4% w/v) from a previously plated glycerol stock and grown overnight at 37°C and 180 rpm. For this culture, we used LB medium with glucose in order to avoid premature aggregation of bacteria encoding the same fimbriae. The following day 5 ml of fresh LB medium were inoculated with 12.5 µl of two different overnight cultures (overall dilution factor of 1:200). The OD_600_ was determined in regular time intervals until an OD_600_ of 0.4–0.6 had been reached, 150 µl of a 10% arabinose solution was added, incubation continued for another 60 min and afterward samples were kept static at room temperature to allow the cells to sediment before microscopic inspection. Samples for microscopy using the inverted microscope Leica DMi8 were taken from the bottom of the tubes. The Leica Application Suite X (LAS X) was used to take and pre-process the images. For further processing, we used ImageJ 1.51 n.

### Biofilm assay

The biofilm-forming capabilities of *E. coli* strains DH5α and 55989 *agg*3^−^ expressing the different AAF variants were tested as described by Reisner and colleagues [29] with the following modifications: the bacterial strains plated on LB agar containing the suitable antibiotics and incubated overnight at 37°C. A single colony was inoculated in 2 ml LB medium containing the suitable antibiotics and grown overnight at 37°C and 180 rpm for 7 h. Afterward, the starter cultures were diluted 1:100 in 3 ml LB medium containing the suitable antibiotics and incubated at 37°C and 180 rpm for 16 h. The cultures were diluted to OD_600_ 0.05 using fresh medium supplemented with arabinose 1% (w/v). Per 96-well plate (clear U-bottom, PVC; Corning, New York, USA), eight wells per strain were filled with 140 µl each, and plates were kept static at 37°C (or 30°C or 20°C, respectively) for 48 h. In order to minimize evaporation, plates were kept in a metal box lined with moist paper towels. Afterward, the wells were washed three times with 200 µl PBS and the plate was dried at 80°C for 1 h. Staining of biofilm was done by incubation with 175 µl crystal violet (CV) (0.1%) per well for 1 h at room temperature. The CV solution was removed, and the wells were washed three times with double distilled H_2_O (ddH2O) and dried for 1 h. For the quantification of biofilm production, each well was treated with 200 µl destaining solution (80% ethanol, 20% acetone) for 30 min. Afterward, 100 µl were transferred to a new 96-well plate and the OD was measured using a Tecan Infinite F200 plate reader at 595 nm. For each strain and condition, we tested three independent biological replicates with six technical repeats each. Data evaluation was done as described by Stepanović and colleagues. The cutoff OD (ODc) was defined as three standard deviations above the mean OD of the negative control values [30]. Therefore, all tested samples shown in Figure 6, except for the bacteria expressing AAF/III and AAF/V, produced significantly more biofilm than the negative control.

### Cell adhesion assay

The cell adhesion assay was done as described by [31], variations summarized by [32], with the following alterations: HEp-2 cells were grown for 48 h at 37°C and 5% CO_2_ until 70–90% confluence in EMEM (Biochrom, Berlin, Germany) in 4-well glass chamber slides (Merck Millipore, Darmstadt, Germany). On the day of the experiment, the cells were washed twice with 1 ml fetal calf serum-free EMEM (FCS-free) and incubated for 3 h at 37°C and 5% CO_2_ with “infection mixture.” The “infection mixture” was produced as follows: five fresh colonies per strain were incubated at 37°C for 16 h under static conditions in EMEM, supplemented with arabinose (final concentration 1%) for induction of AAF expression. OD_600_ was measured the following day and the infection mixture was prepared consisting of 50 µl of 10% mannose (final concentration 0.5%) for blocking type 1 fimbriae [31], the calculated amount of bacterial cells (final concentration 10^8^ cfu/ml) and cell culture medium up to 1 ml. After incubation of HEp-2 cells for 3 h with the infection mixture, the cells were washed thrice with PBS and fixed for 1 min with 1 ml methanol. The methanol was replaced with 1 ml of Giemsa staining solution (freshly diluted 1:20 in ddH_2_O) per well and incubated for 30 min at room temperature. The staining solution was discarded, and the cells were washed carefully with ddH_2_O until the water was clear. The slides were dried at room temperature and cell adherence was evaluated using 40x and 100x oil-immersion light microscopy (Zeiss Axiostar, Carl Zeiss, Jena, Germany). Classification of adherence patterns was done as described in other publications [33,34].

### Electron microscopy

Overnight cultures of EAEC strain 55989 *agg3^−^* carrying one of the plasmids pAAF/I, pAAUF/III, pAAF/IV, pAAF/V, and pBAD24 were diluted in fresh LB medium containing ampicillin (100 µg/ml) and cultivated at 37°C until an OD_600_ of 0.4–0.6 had been reached. Then, 10% arabinose solution was added to a final concentration of 0.3% for the induction of AAF fimbrial expression, and the incubation was continued for another 60 minutes at 37°C. A drop of bacterial culture of each sample was sedimented on a Formvar-coated, carbon-sputtered grid. After negative-staining with 1% phosphotungstic acid, the samples were analyzed at 80 kV with a Tecnai 12 electron microscope (FEI, Eindhoven, The Netherlands). Images of selected areas were documented with Veleta 4k CCD camera (emsis, Münster, Germany).

### Statistical analysis

Statistical comparisons were done by performing a one-way ANOVA with Tukey’s posttest using the software IBM SPSS Statistics Version 25. We set our significance level at *p* < 0.05.

## Results

### Comparison of the gene clusters encoding AAF variants I, III, IV and V

We have previously shown that EAEC strain 55989 expressing AAF/III fimbriae forms smaller aggregates on cultured cells than a Stx2 phage-cured *E. coli* O104:H4 strain expressing AAF/I [24]. This raised the question whether the different types of fimbriae confer phenotypically distinguishable traits that may have added to the exceptionally high virulence of EHEC O104:H4. Figure 1 shows a schematic representation of the genetic organization of the AAF-encoding gene clusters I, III, IV, and V (Figure 1(a)) and a comparison of the amino acid sequence identities of the subunits of AAF/I, IV, and V in respect to the subunits of AAF/III (Figure 1(b)). The amino acid sequences of the chaperones, ushers, and minor subunits showed medium to high amino acid sequence similarity, whereas the major fimbrial subunits were apparently highly variable and showed low to very low similarities. The subunits of the AAF/II fimbriae of prototypic EAEC strain 042 were like the AAF/I subunits of EHEC O104:H4 isolate LB226692 less similar to AAF/III than AAF/V and more similar to AAF/III than AAF/IV with respect to amino acid sequence. They were, however, not encoded in a single operon (data not shown). We therefore decided to focus on AAF variants /I, /III, /IV, and/V and investigated whether AAF/I fimbriae expressed by the EHEC O104:H4 outbreak strain possess different adhesive properties than closely and more distantly related AAF fimbriae of EAEC isolates.

### Cloning and controlled heterologous expression of AAF/I, III, IV, and V in EAEC strain 55989

**Figure 2.**
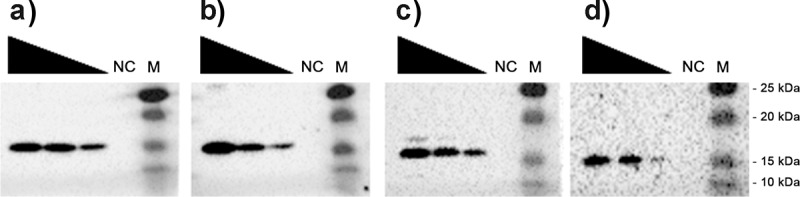
Western blot analysis of EAEC strain 55989 *agg*3^−^ heterologously expressing AAF/I, III, IV and V. Shown is a Western blot analysis of the pBAD24-based expression of the *aaf*/I, III, IV and V determinants. Decreasing amounts of total protein (10 µg, 5 µg and 2.5 µg) of EAEC strain 55989 *agg*3^−^ carrying plasmid pAAF/I (a), pAAF/III (b), pAAF/IV (c) or pAAF/V (d) were used for semi-quantitative Western blot analysis using antibodies raised against each major fimbrial subunit. EAEC strain 55989 *agg*3^−^ carrying the empty vector pBAD24 was used as negative control (NC). Corresponding sizes of immunoreactive bands are given on the right (size marker; M)

Several surface-exposed factors, such as type 1 fimbriae, long polar fimbriae, curli, and cellulose have been described to contribute to biofilm formation and adherence to eukaryotic cells of EAEC strain 55989 [33,34]. In order to be able to directly compare the properties of the AAF fimbriae in a homogeneous genetic background, we first deleted *agg*3*DCBA* from the pAA plasmid of EAEC strain 55989 by Red/ET recombination and cloned the encoding gene clusters for AAF/I, III, IV, and V into the expression vector pBAD24. Afterward, we transformed the plasmids into EAEC strain 55989 *agg*3**^−^**, induced the expression of the AAF fimbriae with arabinose, and confirmed their expression by Western blot analysis with polyclonal antisera raised against major fimbrial subunits of AAF/I, AAF/III, AAF/IV, and AAF/V (Figure 2). A protein concentration-dependent signal of expected size was observed in total protein extracts of EAEC 55989 *agg*3**^−^** containing the AAF/I-, III-, IV- and V encoding expression vectors, but not if EAEC strain 55989 *agg*3**^−^** contained the empty expression vector (negative control, NC). This indicated that the controlled heterologous expression of the subunits of AAF/I, III, IV, and V in EAEC strain 55989 *agg*3**^−^** was successful. As the different AAF allelic variants were heterologously expressed in the isogenic strain background (EAEC 55989 *agg*3-), expression of the aforementioned bacterial factors (type 1 fimbriae, long polar fimbriae, curli and cellulose), that may be involved in bacterial adherence and biofilm formation, is unchanged.

### Confirmation of AAF expression by electron microscopy

**Figure 3.**
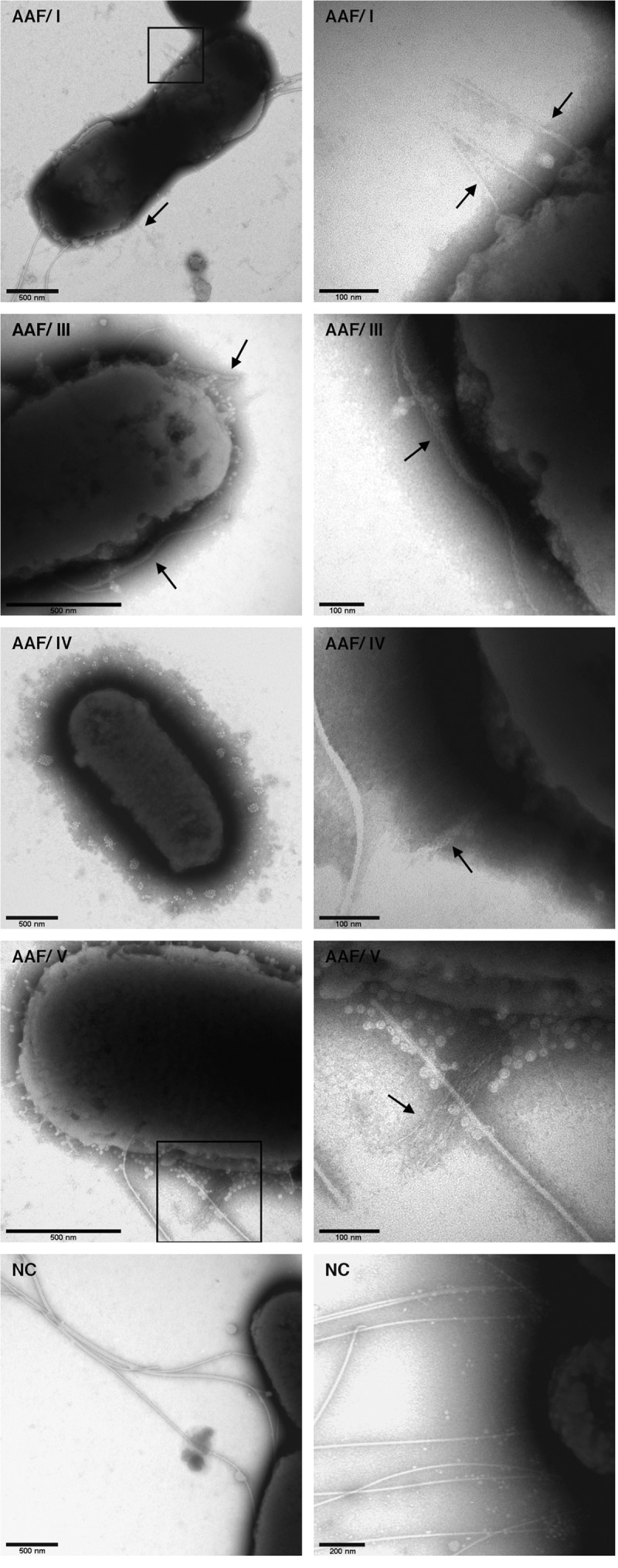
Electron microscopic images of EAEC strain 55989 *agg*3^−^ expressing different AAF variants. Shown are negatively stained EM images of EAEC strain 55989 *agg*3^−^ heterologously expressing AAF/I, III, IV and V (black arrows), as well as EAEC 55989 *agg*3^−^ containing the empty expression vector (NC)

In order to check if the heterologous expression of the AAF subunits also resulted in the expected cell surface structures of host strain EAEC 55989 *agg*3**^−^**, we subjected the bacteria to EM analysis. As shown in Figure 3, thin surface structures resembling the previously described fimbriae of the corresponding wild type cells could be detected when EAEC strain 55989 *agg*3**^−^** was heterologously expressing AAF/I, III, IV, and V, but not when containing the empty plasmid (NC). Irrespective of the plasmid, strain 55989 *agg*3**^−^** was expressing flagella that were substantially longer and thicker than the fimbriae, which were readily distinguishable from the flagella, as expected (Figure 3).

### Sedimentation speed depends on AAF cluster and strain background

We determined the sedimentation kinetics following autoaggregation of EAEC strain 55989 *agg*3^−^ cells expressing the different AAF variants. We induced the expression of AAF/I, III, IV, and V as described above for the preparation of EM samples. Afterward, glass tubes were filled with 5 ml of culture, kept static at room temperature and the cell density was determined directly under the surface of the culture in regular time intervals.

**Figure 4.**
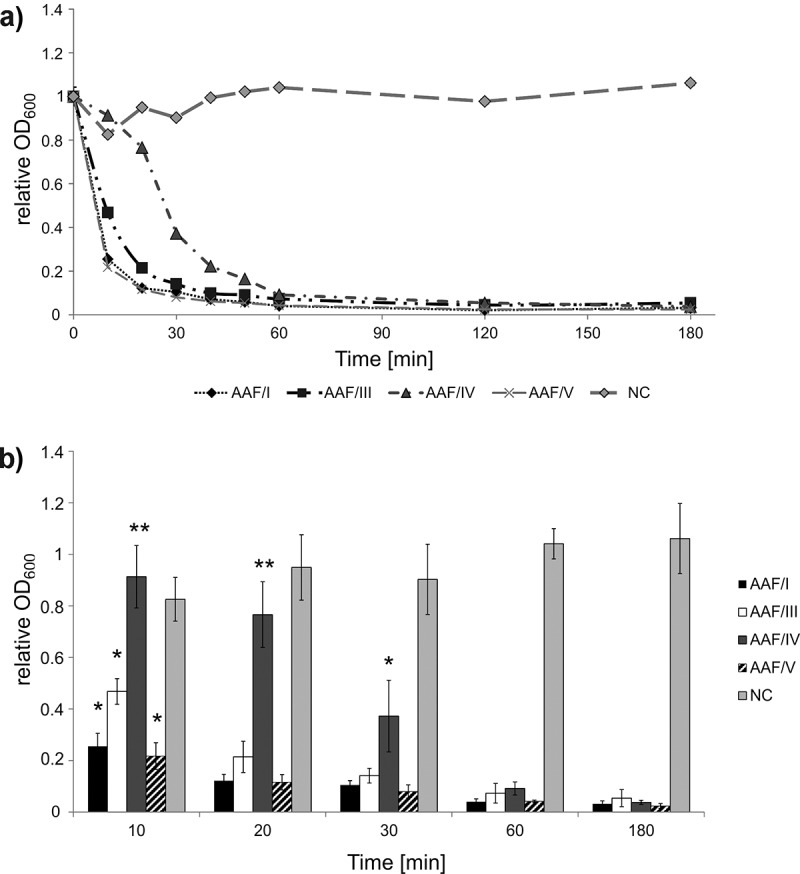
Impact of different AAF variants on bacterial autoaggregation. Shown is the relative decrease in OD_600_ when compared to the OD_600_ at the start of the experiment. Autoaggregation was tested for *E. coli* strain 55989 *agg*3^−^ carrying one of the plasmids pBAD24*aaf*I, pBAD24*aaf*III, pBAD24*aaf*IV and pBAD24*aaf*V or pBAD24 (NC). Samples were taken in ten min intervals until reaching a plateau. (a) Representative results of an autoaggregation assay. Except for *E. coli* strain 55989 *agg*3^−^ expressing AAF/IV, the bacterial culture sedimented completely at t = 60 min. (b) Shown are average values for each time point and standard deviations of three biological replicates. Whereas the sedimentation of AAF/I-, AAF/III- and AAF/V-expressing cells differed significantly (p < 0.05) from the negative control already after 10 min, AAF/IV-expressing cells differed only after 30 min significantly from the negative control (the first time point at which the sedimentation speed is significantly different from the negative control is indicated with an * for each AAF variant). The sedimentation speed of AAF/IV-expressing cells was significantly different from AAF/I-, AAF/III- and AAF/V-expressing bacteria until t = 20 min (indicated with **)

Figure 4(a) shows the results of a typical assay. The bacterial autoaggregation was strictly dependent on the expression of the AAF-encoding gene clusters as judged by complete absence of sedimentation for the negative control, for which the OD_600_ slightly increased, as expected (compare AAF/I, AAF/III, AAF/IV, and AAF/V to vector control in Figure 4(a)). Amongst the AAF variants, AAF/I and AAF/V mediated reproducibly the fastest sedimentation with a comparable rate, followed by AAF/III, which lagged until t = 20 min (Figure 4(b)). Of all tested fimbrial variants, expression of AAF/IV resulted in the slowest bacterial sedimentation and did so significantly until t = 30 min (p < 0.001). After 60 min, no further significant drop in OD_600_ was observable.

To test if antibodies raised against the different AAF variants inhibit the sedimentation phenotype, we added suspensions of purified antibodies raised against the different AAF subtypes to EAEC strain 55989 *agg*3^−^ expressing one of the AAF/I, AAF/III, AAF/IV, or AAF/V fimbriae (Figure S1). The antibodies completely prevented sedimentation of EAEC strain 55989 *agg*3^−^ expressing AAF/I and AAF/IV. For EAEC 55989 *agg*3^−^ expressing AAF/III, sedimentation was markedly impeded. For EAEC 55989 *agg*3^−^ expressing AAF/V, the sedimentation was not affected upon addition of the α-AAF/V antibody. However, as the antibody for AAF/V was also requiring substantially more time for the detection of the AAF/V signal in the Western blot analysis (Figure 2d) we assume that the concentration of the antibody stock solution was in this case too low for a complete block. In summary, the different heterologously expressed AAF variants promote bacterial aggregation and sedimentation to varying degrees. If the interaction of the AAF among each other is disturbed, e.g. by adding AAF-specific antibodies, sedimentation is inhibited.

### Mixed autoaggregation assay

**Figure 5.**
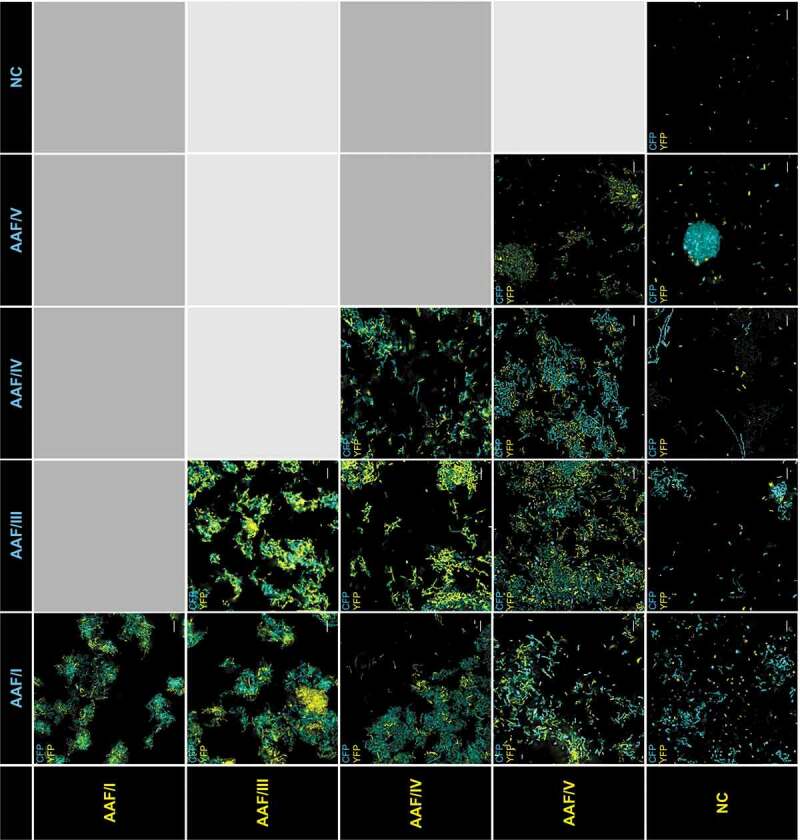
Autoaggregation of mixed EAEC 55989 *agg*3^−^ populations expressing different AAF variants. Shown are fluorescence microscopic images of aggregates formed by EAEC strain 55989 *agg*3^−^ heterologously expressing the same, different or no AAF operon. In order to be able to distinguish the bacteria expressing the different AAF subtypes, the bacteria carried an additional identical plasmid, except for the encoded fluorophore (YFP, yellow/CFP, cyan). The bacterial cells formed more or less homogeneous aggregates, but only upon expression of AAF

The well-known AAF-dependent stacked-brick adherence pattern formed by EAEC on epithelial cells suggested that the fimbriae do not only interact with receptors on eukaryotic cells, but also with each other. However, it was unclear, if these are specific interactions in between the same AAF fimbrial subtype, in between AAF fimbriae in general, or if the fimbriae can also bind to non-AAF surfaces on the bacterial cell wall. In order to analyze the composition of the bacterial aggregates when *E. coli* strain 55989 *agg*3^−^ expressed different AAF subtypes, we mixed two strains marked with otherwise identical plasmids that either expressed the yellow fluorescent protein (YFP) or the cyan fluorescent protein (CFP). Afterward, we induced the expression of the different AAF variants as described above. This allowed us to microscopically analyze the resulting EAEC aggregates (Figure 5). As expected, EAEC strain 55989 *agg*3^−^ containing the empty vector did not form any aggregates whereas homogeneously mixed aggregates were formed when the bacteria were expressing the same AAF subtype with different fluorophores (Figure 5 right bottom to left top). The combination of EAEC 55989 *agg*3^−^ expressing AAF/I, AAF/III, AAF/IV, and AAF/V with the identical host strain containing the empty expression vector did not result in the systematic inclusion of planktonic bacteria into the aggregates (Figure 5, far right panels). When bacteria expressing the different AAF subtypes were mixed together, less homogenously mixed aggregates were formed with rather patchy characteristics, in which cells expressing one AAF subtype appeared to cluster within the aggregates, e.g. for AAF/I and AAF/III (Figure 5). Interestingly, EAEC strain 55989 *agg*3^−^ expressing AAF/V was often found to form dense spherical aggregates that were not observed when the same host strain expressed the other AAF subtypes (Figure 5).

### Impact of individual AAF variants on bacterial biofilm formation

Due to the genetic heterogeneity of EAEC and potential differences in the regulation and expression strength of the different AAF-encoding gene clusters, it was not possible to directly test whether the specific AAF subtype of wild-type isolates affects biofilm formation. We therefore compared biofilm formation upon heterologous expression of the different AAF constructs in an isogenic EAEC 55989 *agg*3^−^ background at 37°C, 30°C, and 20°C in a standard biofilm assay.

**Figure 6.**
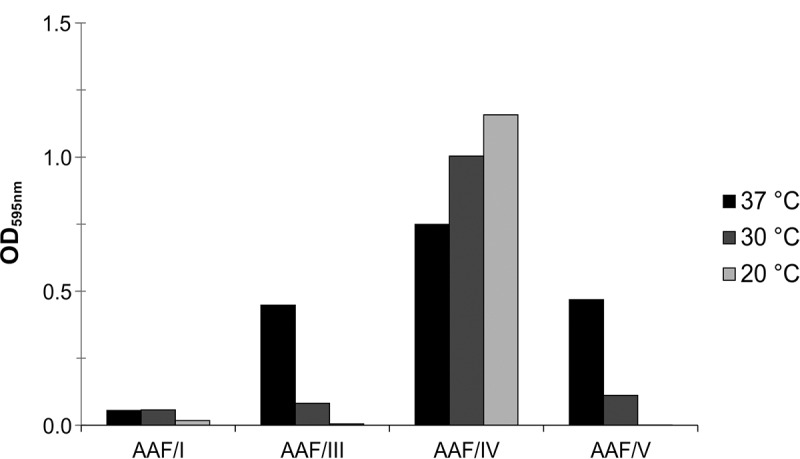
Biofilm formation by EAEC strain 55989 *agg*3- expressing AAF/I, AAF/III, AAF/IV and AAF/V depends on the AAF subtype and temperature. Shown is the biofilm formation in LB medium at 20°C, 30°C and 37°C. For details see text

Interestingly, EAEC strain 55989 *agg*3^−^ expressing AAF/I was not able to form biofilms at any tested temperature. AAF/IV-expressing EAEC strain 55989 *agg*3^−^ produced the most biofilm at 37°C, followed by AAF/V- and AAF/III-expressing strains (Figure 6, black bars). Biofilm formation by AAF/III and AAF/V was generally reduced at lower temperature (compare light gray bars in Figure 6). Expression of AAF/IV fimbriae resulted in the highest levels of biofilm formation at all tested conditions.

### Cell adhesion

**Figure 7.**
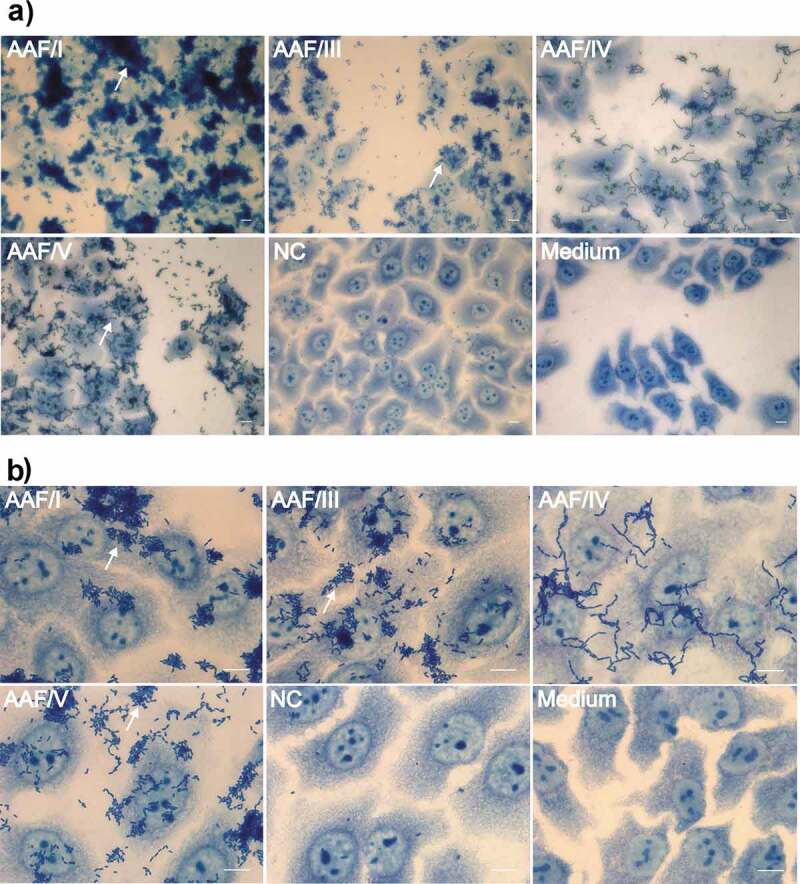
Impact of AAF subtype expression on the aggregative adherence phenotype. Shown are bacterial aggregates formed by EAEC strain 55989 *agg*3^−^ heterologously expressing AAF/I, AAF/III, AAF/IV and AAF/V on HEp2 cells as indicated. NC: negative control (pBAD24); Medium: uninfected HEp2 cells; Scale bar: 10 µm; Upper panels: 10x magnification, Lower panels: 40x magnification. White arrows indicate rudimentary honey comb formation

Adhesive properties of the different AAF variants were tested on HEp-2 cells (Figure 7), which is the current gold standard for testing cell adhesion patterns of EAEC [35]. EAEC strain 55989 *agg*3^−^ expressing AAF/I, AAF/III, or AAF/V formed a similar pattern. The bacteria piled up to stacked bricks, which resembled the aggregative adherence (AA) pattern as previously described [36]. Notably, expression of the AAF/I variant also led to rudiments of honeycomb formations and resulted in the formation of the largest aggregates on the cells, very similar to what has been described for EHEC O104:H4 (Figure 7, 40x magnification) [24].

The EAEC strain 55989 *agg*3^−^ expressing AAF/IV displayed a variation in its adherence pattern. Especially noticeable in less densely occupied areas of the eukaryotic cells the bacteria formed a serpentine line pattern, which occasionally branched off or less frequently run parallel to one another. Overall, EAEC strain 55989 *agg*3^−^ expressing AAF/IV formed stacked-brick like patterns only when higher local concentrations of bacteria had been reached.

## Discussion

The hypervirulent German outbreak strain EHEC O104:H4 encoded AAF/I, whereas EAEC strain 55989, one of its closest known relatives, encoded AAF/III [9]. The combination of a Stx2-encoding prophage with typical EAEC virulence markers was proposed to have resulted in the unusually aggressive phenotype and we had previously observed that Stx2 phage-cured EHEC O104:H4 formed larger aggregates than other tested EAEC strains on eukaryotic cells *in vitro* [24]. Against this background, we wanted to gain insights into phenotypic differences mediated by AAF/I and AAF/III. In order to assess potential differences, we decided to use closely and more distantly related reference fimbriae for a phenotypic comparison (Figure 1). The heterologous expression of the AAF gene clusters in comparable amounts in EAEC strain 55989 *agg*3*^−^* was successful, and surface-exposed fimbrial structures were detected resembling equivalent AAF fimbriae of previous studies [5,13–15,37]. Due to a heavily stained matrix around the cells, the imaging of AAF expression in the EAEC 55989 strain background was very challenging. We anticipate that capsular polysaccharide or colanic acid is the main component of this matrix as large amounts of shedded “slime” blebs can be seen on the bacterial cell surface (Figure 3).

As the ability to aggregate is considered to be an important trait for bacterial survival during host infection [38], we compared the aggregative behavior between the different AAF-expressing strains. Bacterial cells expressing AAF/I autoaggregated and showed a very fast sedimentation behavior. Although faster than the AAF/IV expressing strain it was comparable to AAF/III- and IV-expressing isogenic strains, indicating no special aggregation property. Less efficient sedimentation by AAF/IV-expressing bacteria may not necessarily be due to a faster formation of the aggregates, but could as well be due to differences in shape and size of the aggregates. The disturbance of the interaction between AAF on the bacterial surface abolished or decreased bacterial sedimentation.

We further investigated the composition of the bacterial aggregates that are formed when the same, different, or no AAF fimbriae were expressed by EAEC strain 55989 *agg*3*^−^* (Figure 5). It might appear obvious that only bacterial cells that express AAF are integrated into the aggregates. However, the interactions of the highly positively charged major fimbrial subunits with known receptors, e.g. fibronectin and the transmembrane mucin MUC1, were proposed to be solely based on electrostatic interactions and therefore rather unspecific [39]. Furthermore, dispersin was originally suggested to counteract the hyperaggregative phenotype of *aap*-negative bacteria by preventing the collapse of the positively charged fimbriae on the negatively charged bacterial surface [40]. Such electrostatic interactions of the heterologously expressed fimbriae with the cell surface of the AAF-negative isogenic strain were therefore anticipated. In this context, we further observed that bacteria formed mixed aggregates when combinations of different AAF were expressed, albeit generally less homogeneous as bacteria expressing the same AAF subtype. This suggests that AAF mediate specific bacteria–bacteria interactions, which may be mediated by the minor fimbrial subunit. This assumption is supported by the very well mixed aggregates formed by AAF/III and AAF/V that possess identical minor subunits (Figure 1) as opposed to rather patchy types of aggregates formed by, e.g., AAF/III- and AAF/I-expressing bacteria (Figure 5). In order to clarify this question, it will be necessary to analyze the aggregates formed by bacteria that express hybrid AAF operons.

With respect to the biofilm formation capacities, bacteria expressing AAF/I were not or much less efficient in biofilm formation on PVC (especially prominent at 37°C), compared to the closely related AAF/III and AAF/V-expressing strains, which formed intermediate biofilms in a temperature-dependent manner. In contrast, high biofilm formation was observed with bacteria expressing AAF/IV, which is supported by the fact that also wild type EAEC isolates encoding this cluster were described to adhere quite strongly to glass surfaces and to form strong biofilms [11,12].

Overall we found that the sedimentation speed following autoaggregation of bacteria expressing different AAF variants was apparently anti-correlated with the capacity to form biofilms on PVC (compare Figures 4 and 6). This might appear paradox at first sight, but if, e.g., the affinities of the fimbriae toward each other are higher than the affinity toward the surface, the bacterial aggregates might simply form too fast and sediment before an effective attachment to the surface can occur.

AAF/IV also appeared phenotypically distinct on HEp-2 cells when compared to the adherence phenotype of EAEC strain 55989 *agg*3*^−^* expressing the other AAF variants (Figure 7). AAF/IV expression resulted in a chain-like bacterial adherence pattern to HEp-2 cells. This head-to-tail like bacterial interaction was apparently not dependent on contact with eukaryotic cells, as it could be also observed for AAF/IV-expressing EAEC strain 55,989 *agg*3*^−^* in the mixed autoaggregation assay (e.g. Figure 5, AAF/IV in combination with negative control). This suggests that AAF/IV may differ functionally from the other AAF subtypes and may have a function outside of the human host. AAF/IV was also referred to as Hda (HUS-associated diffusely adherent) originating from the plasmid pO86A1 of clinical isolate DIJ1 (GenBank accession no. AB255435.1). Although this chain-like adherence phenotype of some EAEC variants, especially in correlation with AAF/IV expression, has been reported before [41,42], the exact phenotype mediated by this particular subtype of AAF still deserves further investigation. Nonetheless, it is interesting to note, that in a study on the impact of EAEC infection on childhood malnutrition and inflammation the presence of the AAF/IV, in contrast to the other AAF subtypes, was significantly associated with nourished children [43].

In contrast to that, AAF/I, AAF/III, and AAF/V showed aggregates that were partially reminiscent of the strictly two-dimensional “stacked-brick” pattern described for the corresponding wild type cells [24] (Figure 7, note loci with less dense bacterial aggregates). However, most of the formed bacterial aggregates were rather three-dimensional, which may have disturbed the formation of a regular two-dimensional arrangement. Notably, the bacteria expressing AAF/I formed the largest aggregates on HEp-2 cells (Figure 7), as previously observed for the corresponding EHEC O104:H4 outbreak strain [24]. Thus, this appears to be a trait that is mediated directly by this specific AAF subtype.

The aggregative adherence phenotype can also be conferred by another adhesin designated aggregate-forming pilus (AFP). AFP is more similar to the bundle-forming pilus (BFP), which is usually expressed in enteropathogenic *E. coli* (EPEC), in terms of their operon structure and relatedness of their protein subunits. Expression of the *afp* operon in AAF-negative EAEC/EHEC hybrids has been correlated with aggregative adherence [44]. It will thus be interesting to compare AAF and AFP with regard to their individual contribution to aggregative adherence and biofilm formation.

Our comparative analysis of AAF/I, III, IV, and V-expressing EAEC strains confirmed that the adherence and aggregation phenotype mediated by AAF/V is more similar to that of AAF/III than that of AAF/I. We show for the first time that these AAF subtypes mediate bacteria–bacteria interactions, which exclusively affect bacterial cells expressing AAF. This raises questions about the exact role of AAF in aggregative adherence to epithelial cells, in particular its primary involvement in bacterial autoaggregation and interaction with host factors. Moreover, our work indicates that the strong aggregative adherence phenotype conferred by AAF/I compared to other AAF subtypes is very likely less dependent on differences in expression and/or regulation of AAF, but rather on the structural fimbrial proteins themselves [24]. Hence, the combination of AAF/I of EAEC with a *stx*2-encoding prophage from EHEC in one single *E. coli* bacterium might truly have been the “worst of both worlds” that we have so far experienced.

## Supplementary Material

Supplemental Material
