# Supplementary material for: The Superior Adherence Phenotype of E. coli O104:H4 is Directly Mediated by the Aggregative Adherence Fimbriae Type I
Source: Virulence. 2021 Jan 15;12(1):346–59. doi: 10.1080/21505594.2020.1868841 (PMC7834096; doi:10.1080/21505594.2020.1868841)
Supplement: Supplemental Material [file KVIR_A_1868841_SM6035.docx]

# The Superior Adherence Phenotype of *E. coli* O104:H4 is Directly Mediated by the Aggregative Adherence Fimbriae Type I

Philipp Schiller^a#^, Michael Knödler^a#^, Michael Berger^a^, Petya Berger^a^, Lilo Greune^b^, Angelika Fruth^c^, Alexander Mellmann^a^, Petra Dersch^b^, Ulrich Dobrindt^a^*

^a^Institute of Hygiene, University of Münster, 48149 Münster, Germany

^b^Institute for Infectiology, University of Münster, 48149 Münster, Germany

^c^Robert Koch Institute, Division of Enteropathogenic Bacteria and Legionella, 38855 Wernigerode, Germany

(^#^both authors contributed equally to this work)

## Supplementary Material

**Supplementary Table S1:** Bacterial strains and plasmids used in this study

| Strain | Reference or origin |
| --- | --- |
| LB226692 | (Bielaszewska et al., 2011) |
| EAEC 55989 | (Bernier et al., 2002) |
| 13-00093 | EAEC (O73:H18) |
| 147/06 (alternative designation: 1352) | (Toval et al., 2014) |
| DH5α | (Bethesda Research Laboratories, 1986), Thermo Fisher, Germany |
| DH5α (pBAD24) | This study |
| DH5α (pBAD24*aaf*I) | This study |
| DH5α (pBAD24*aaf*III) | This study |
| DH5α (pBAD24*aaf*IV) | This study |
| DH5α (pBAD24*aaf*V) | This study |
| EAEC 55989 *agg*3^-^ | This study |
| EAEC 55989 *agg*3^-^ (pBAD24*aaf*I) | This study |
| EAEC 55989 *agg*3^-^ (pBAD24*aaf*III) | This study |
| EAEC 55989 *agg*3^-^ (pBAD24*aaf*IV) | This study |
| EAEC 55989 *agg*3^-^ (pBAD24*aaf*V) | This study |
| EAEC 55989 *agg*3^-^ (pBAD24) | This study |
| EAEC 55989 *agg*3^-^ (pBAD24*aaf*I) (pPS1) | This study |
| EAEC 55989 *agg*3^-^ (pBAD24*aaf*III) (pPS1) | This study |
| EAEC 55989 *agg*3^-^ (pBAD24*aaf*IV) (pPS1) | This study |
| EAEC 55989 *agg*3^-^ (pBAD24*aaf*V) (pPS1) | This study |
| EAEC 55989 *agg*3^-^ (pBAD24) (pPS1) | This study |
| EAEC 55989 *agg*3^-^ (pBAD24*aaf*I) (pPS2) | This study |
| EAEC 55989 *agg*3^-^ (pBAD24*aaf*III) (pPS2) | This study |
| EAEC 55989 *agg*3^-^ (pBAD24*aaf*IV) (pPS2) | This study |
| EAEC 55989 *agg*3^-^ (pBAD24*aaf*V) (pPS2) | This study |
| EAEC 55989 *agg*3^-^ (pBAD24) (pPS2) | This study |

**Supplementary Table S2:** Plasmids used and generated in this study.

| Plasmids | Origin or Reference |
| --- | --- |
| pBAB24 | Guzman et al., 1995) |
| pBAD24*aaf*I | This study |
| pBAD24*aaf*III | This study |
| pBAD24*aaf*IV | This study |
| pBAD24*aaf*V | This study |
| pKD3 | Datsenko and Wanner, 2000 |
| pKD46 | Datsenko and Wanner, 2000 |
| pWKS30 | Wang and Kushner, 1991 |
| pPS1 | This study |
| pPS2 | This study |

**Supplementary Table S3:** Primer used during in this study.

| Primer | Sequence (5’ – 3’) |
| --- | --- |
| agg_for | GGGGTACCCCAAGTTCAAGTGATAGCGATG |
| agg_rev | GGGGTACCCCAAGAGTTAATTCCCAGAGTG |
| agg3_for | GCTCTAGAGCATGACAAAATCTGTATCAACC |
| agg3_rev | CCCAAGCTTAGCGCACTGTTTTTA |
| hda_for | CTAGCTAGCTAGAGGAAAGAGGGTAGTGACAA |
| hda_rev | CCCAAGCTTGGGACAATGAAAAAGGGATGATG |
| agg5_for | GCTCTAGAGCATAGTGGACGTGGCTAAGTG |
| agg5_rev | GCTCTAGAGCGAGCCTGTAACTGTTTTTCG |
| MBP256 | GCACTATATACTCTATATTAGAAATGCATATGACTACCCTAAGAAAATATATGGG  AATTAGCCATGGTCC |
| MBP257 | ACTTATGCGATCAAAAAGCGCTAAGTGGGGCTTAGCGCACTGTTTTTAATGTGTAG  GCTGGAGCTGCTTC |
| MBP258 | TGAATACAACAGTACTGCGA |
| MBP259 | GACGACTCGGTGAAAAAAG |
| MBP260 | CGAAGTGATCTTCCGTCACA |
| MBP261 | CTGATGATATTTTTCCCTGA |
| MBPD 82 | AACTGCAGCGCTGAGGTCTGCCTC |
| MBPD 83 | GGGGTACCGTCCCGTCAAGTCAG |
| MBPD 132 | TTAATCTCGTTAATTACTGGGACATAACATCAAGAGGATATGAAATTATGGTGTCTATCACTAAAGATC |
| MBPD 133 | AGGAAGCCGCTTTTATCGGGTACTAAAGTTCTGCACCATCAGCGATGGATAGGAAACAGCTATGACCATG |
| MBPD 156 | GGCTGTCGCTATTCTCTCG |
| MBP 140 | CAACACAGTGATTATTTAGCG |

**Supplementary** **Table S4:** Antibodies used in this study.

| Antibody type | Target protein | Host  organism | Peptide sequence  (derived from) | Source |
| --- | --- | --- | --- | --- |
| primary  (polyclonal) | α-AggA | rabbit | QHAKVAKQKYTLNPSIDGGAD  (STEC LB226692) | Davids Biotech (Germany) |
| primary  (polyclonal) | α-Agg3A | rabbit | AADPVITGNKGNIASATAKLK  (EAEC 55989) | Davids Biotech (Germany) |
| primary  (polyclonal) | α-HdaA | rabbit | KTTAKNDELWGYLELTQT  (EAEC 13-00093) | Davids Biotech (Germany) |
| primary  (polyclonal) | α-Agg5A | rabbit | ADAKDGKLVMVKGNDRYTL  (aUPEC 147/06) | Davids Biotech (Germany) |
| secondary  (monoclonal) | α-rabbit  IgG-HRP | goat | - | Dianova (Germany) |


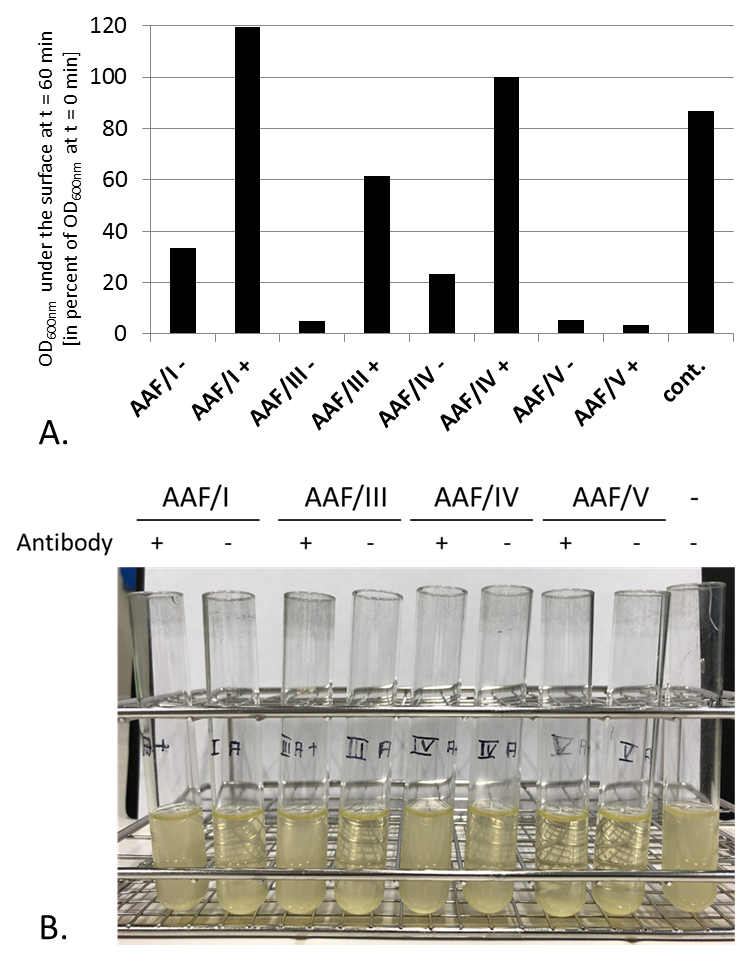


**Figure S1: Inhibition of bacterial sedimentation by antibodies raised against different AAF variants.** To test if antibodies raised against the AAF variants disturb the interaction between AAF on the bacterial surface, thus inhibiting bacterial sedimentation, we added suspensions of purified antibodies raised against the different AAF subtypes to EAEC strain 55989 *agg*3^-^ expressing one of the AAF/I, AAF/III, AAF/IV or AAF/V fimbriae. After 60 min of incubation at room temperature, the sedimentation state of the bacterial cultures was documented. Addition of antibodies completely prevented sedimentation of EAEC strain 55989 *agg*3^-^ expressing AAF/I and AAF/IV. For EAEC strain 55989 *agg*3^-^ expressing AAF/III, sedimentation was markedly impeded. Sedimentation of EAEC strain 55989 *agg*3^-^ expressing AAF/V was not affected upon addition of the AAF/V antibody, probably because the concentration of the antibody stock solution was in this case too low for efficient interference with AAF interaction. EAEC strain 55989 *agg*3^-^ (pBAD24) was used as a negative control (rightmost tube).
